# Efficiency of Event-Based Sampling According to Error Energy Criterion

**DOI:** 10.3390/s100302242

**Published:** 2010-03-18

**Authors:** Marek Miskowicz

**Affiliations:** Department of Electronics, AGH University of Science and Technology, al. Mickiewicza 30, 30-059 Kraków, Poland; E-Mail: miskow@agh.edu.pl; Tel.: +48 12 6173034; Fax: +48 12 6332398

**Keywords:** event-based sampling, send-on-delta, sampling error

## Abstract

The paper belongs to the studies that deal with the effectiveness of the particular event-based sampling scheme compared to the conventional periodic sampling as a reference. In the present study, the event-based sampling according to a constant energy of sampling error is analyzed. This criterion is suitable for applications where the energy of sampling error should be bounded (*i.e.*, in building automation, or in greenhouse climate monitoring and control). Compared to the integral sampling criteria, the error energy criterion gives more weight to extreme sampling error values. The proposed sampling principle extends a range of event-based sampling schemes and makes the choice of particular sampling criterion more flexible to application requirements. In the paper, it is proved analytically that the proposed event-based sampling criterion is more effective than the periodic sampling by a factor defined by the ratio of the maximum to the mean of the cubic root of the signal time-derivative square in the analyzed time interval. Furthermore, it is shown that the sampling according to energy criterion is less effective than the send-on-delta scheme but more effective than the sampling according to integral criterion. On the other hand, it is indicated that higher effectiveness in sampling according to the selected event-based criterion is obtained at the cost of increasing the total sampling error defined as the sum of errors for all the samples taken.

## 1. Introduction

Due to demand of developing low power wireless devices and systems, a growing interest in investigating event-based sampling schemes has been observed in recent years. On the system level, the representative example of event-based sampling application is a wireless sensor network that is essentially an event-based system intended to detect specified events of interest in a sensor field [1,2]. On the device level, event-based sampling has been applied in *asynchronous analog-to-digital converters* (A-ADCs) which are a new class of converters proposed recently [3–7]. In the A-ADCs, the periodic sampling is substituted by *signal-dependent* schemes where the sampling operations are triggered irregularly if a signal value varies by a specified increment. The A-ADCs are clockless circuits designed for ultra low-power and low-voltage sensing devices.

The event-based schemes are attractive especially for sampling *bursty signals* [1,7–9]. As known, many signals in sensory applications (e.g., temperature sensors, speech signals, electrocardiograms, *etc.*) show bursty statistical properties. Bursty signals are constant for most of the time and may vary significantly only during short time intervals.

The idea of varying the sampling period and adjusting it to the current signal behavior is not new, see e.g., [10]. Since the early 60s, the *adaptive sampling*, that belongs to the class of signal-dependent techniques closely related to the event-based schemes, have been developed [11–18]. The adaptive sampling schemes are based on the real-time adjustment of the temporary sampling period to the predicted signal changes. The sampling period is allowed to vary from interval to interval in order to reduce the number of samples without degrading a system response. On the other hand, in several studies, the sampling period is adapted in real-time to achieve a satisfactory performance of a networked sensor/control system in varying load conditions [19], or of an embedded system with scheduling a set of controller tasks [20].

The essential difference between the adaptive sampling and the event-based sampling is that the former one is based on the *time-triggered* strategy where although the sampling instants are still controlled by the timer, the intersampling intervals may change. The event-based sampling, on the other hand, belongs to the *event-triggered* systems where the sampling operations are determined only by signal amplitude variations rather than by the progression of time.

Thus, the *event-based sampling* schemes belong to a special class of irregular observations where a pre-specified functional relationship between the sampling instants and signal behavior occurs. This relationship is defined by the sampling criterion. More specifically, in the event-based sampling, the signal is sampled when the *significant event* occurs (*i.e.*, a significant change of its parameters is noted) [20]. Event-based data collection is used in the *reactive networks* where the sensing devices send new reports only when the variable being monitored increases or decreases beyond a threshold [21].

Various event-based sampling criteria have been proposed in the scientific literature in the past. In particular, numerous contributions to the state of the art in event-based sampling have been published in *Sensors* journal [1,2,8,9,19,22,23,26,27]. The most natural signal-dependent sampling scheme is the send-on-delta principle [1,29,30–32]. According to this scheme the sampling is triggered if the signal deviates by *delta* defined as a *significant change* of its value referred to the most recent sample. The efficiency of the send-on-delta concept compared to the periodic sampling has been presented in [1]. Many studies that deal with the send-on-delta sampling use different terminology to describe this sampling principle. The term *send-on-delta* is accommodated to define data reporting strategy in sensor networking [1,9,29–32,40,42]. In the control systems community, the name the *deadband sampling* [24,36,41], or the *Lebesgue sampling* [33] is applied. On the other hand, the term *level-crossing sampling* is used in the context of signal conversion and processing [3,4,6,7,34–35].

The modified send-on-delta reporting strategy that improves network estimation performance when packet dropouts happen is proposed in [22]. A new sampling criterion, the *send-on-delta* scheme *with prediction*, that makes use of the first-order signal value prediction to trigger sampling operations has been introduced and compared to the pure send-on-delta technique in [9]. In References [8,23], the integral criteria for extensions of the linear send-on-delta scheme have been studied. The integral criterion introduced in [8] is suitable for tracking the signal in case of occurrence of the steady-state error but is sensitive to noise. The criterion introduced in [23], called the *area-triggered sampling*, operates well in noisy environments. On the other hand, it may be shown that the asymptotic effectiveness of the integral criterion in relation to the periodic sampling derived in [8] is valid also for the area-triggered sampling scheme proposed in [23]. The comparison of various event-based sampling schemes in particular applications is analyzed in [24–26] as follows. In [26], the characteristics of several event-based sampling schemes based on the experimental results of the level control of a tank is carried out. The relevance of particular event-based sampling schemes in the context of intelligent building networked control systems is presented in [25]. The application of the event-based sampling to greenhouse climate monitoring and control is reported in [2]. The further research problems referred to the systems with aperiodic sampling (which the event-based indeed is, among others) are studied in [27,28]. In [27], the problem of choosing sampling and hold devices to achieve output deviations close to its sampled values is analyzed. On the other hand, the properties of reachability, observability, controllability, and constructability of discrete-time linear time-invariant dynamic systems when the sampling instants are chosen aperiodically are discussed in [28].

A parallel line of research concerns the event-based control that has emerged as an attractive approach for addressing the problem of control system design under rate limitations [37–43].

The present paper is a next step in the series of the author’s studies [1,8] that deal with the effectiveness of the particular event-based sampling scheme compared to the conventional periodic sampling as a reference. In this study, the analysis of the event-based scheme according to a constant *energy of sampling error* is presented. In general, this criterion, proposed in [45], is suitable for applications where the energy of sampling error should be bounded. This criterion may be also considered as *variance-based* since the continuous-time signal is sampled when the variance of the sampling error referred to the most recent sample reaches a predefined threshold.

In the error energy sampling criterion similarly as in the integral criteria, the sampling time is a function of the intersampling signal behavior since the signal variations occurring within the intersampling interval are successively accumulated. Instead, in the send-on-delta scheme, the sampling time depends only on the instantaneous signal value deviation by delta referred to the most recent sample [1]. The difference is that the successive sampling error values are squared before integration in the error energy based sampling criterion. Compared to the integral sampling criteria [8] (and area-triggered sampling [23]), the error energy criterion gives more weight to extreme sampling error values.

The present paper is an extended version of [45]. In the studies [1,8] as well as in the present paper, the comparison of both schemes is carried out for equality of the maximum sampling error in the domain that corresponds to the particular event-based sampling criterion. Thus, the maximum linear sampling error has been used for a comparison of the send-on-delta scheme and the periodic sampling [1]. To compare the event-based sampling according to the integral criterion to the conventional periodic sampling, the maximum integral error has been selected [8]. Consequently, in the present paper the comparison is carried out for the same maximum energy of the sampling error. Furthermore, the generalized criterion for event-based sampling is formulated and the concept of event-based temporal sampling has been extended to sampling signals in the space domain.

The paper contribution consists essentially in providing an analytical approximation of the mean sampling rate, and the derivation of the asymptotic effectiveness of the sampling according to the error energy criterion, which is defined as the effectiveness for an infinite sampling resolution. As it will be shown on the basis of the exemplified results, the effectiveness of the sampling according to the energy criterion for finite resolution differs not much from the asymptotic effectiveness. The applications of the derived formula for common transient responses in dynamical systems is presented. Finally, the asymptotic effectiveness of the sampling according to energy criterion is compared to the effectiveness of the send-on-delta, and of the event-based integral sampling.

## 2. Event-Based Sampling according to Energy Criterion

### 2.1. Sampled Signal Definition

Let us assume that a sampled signal belongs to a class of signals of *bounded variation*. A signal *x*(*t*) defined over a time interval [*a*,*b*] is said to be of bounded variation if the following sum is bounded [46,47]:

(1) $$\sum_{j=1}^{k}\left|x(t_{j})-x(t_{j-1})\right|<\infty$$

for every partition of the interval [*a*,*b*] into subintervals (*t*_*j*−1_, *t_j_*), where *j* = 1,2…,*k*; and *t*_0_ = *a*, *t_k_* = *b*. The measure denoted by $V_{a}^{b}(x)$ and defined as:

(2) $$V_{a}^{b}(x)\overset{\mathrm{def}}{=}\text{sup}\sum_{j=1}^{k}\left|x(t_{j})-x(t_{j-1})\right|$$

is said to be a *total variation* of a signal *x*(*t*) on the interval [*a*,*b*].

**Figure 1.**
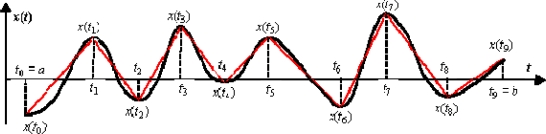
An example of a signal of bounded variation on the interval [*a*,*b*] where a partition of [*a*,*b*] into a number of *k* = 9 subintervals is arranged. The vertical component (ordinate) of the length of the broken line corresponds to the total variation $V_{a}^{b}\left(x\right)$ of the signal *x*(*t*) on the interval [*a*,*b*].

A signal of bounded variation is not necessarily continuous but it is differentiable almost everywhere. Moreover, *x*(*t*) can have discontinuities of the *finite-jump* type at most [46,47]. If *x*(*t*) is continuous, the total variation $V_{a}^{b}\left(x\right)$ might be interpreted as the vertical component (ordinate) of the arc-length of the *x*(*t*) graph, or alternatively, as the sum of all consecutive peak-to-valley differences. The definition of the signal of bounded varation is exemplified in Figure 1 where the partition of the interval [*a*,*b*] into a number of *k* = 9 subintervals is arranged, and the graphical interpretation of $V_{a}^{b}\left(x\right)$ is illustrated.

All the signals which appear in the physical reality or are generated in the laboratory can be approximated by a class of signals of bounded variations.

### 2.2. Definition of Energy Criterion

By the definition, a signal *x*(*t*) is sampled at the instant *t_i_* according to the energy criterion if the energy of a difference between the signal value *x*(*t_i_*) and the value included in the most recent sample *x*(*t*_*i*−1_) accumulated during the *i*th sampling interval (*t*_*i*−1_,*t_i_*) reaches a certain constant threshold *ζ* > 0:

(3) $$\int_{t_{i-1}}^{t_{i}}{\left[x(t)-x(t_{i-1})\right]}^{2}\;\mathrm{dt}=\zeta$$

where *i* = 1,2,…,*n* is a number of samples taken during the analyzed time interval [*a*,*b*].

We assume also that the zero-order hold is used to keep the value of the most recent sample between sampling instants.

A detection of events that trigger sampling operations according to the error energy criterion may be implemented in the analog circuit, or using digital processing where the continuous-time signal is represented by periodic samples taken with high frequency.

The former implementation needs a circuit squaring the difference between the instantaneous signal value *x*(*t*) and the value corresponding to the most recent sample *x*(*t*_*i*−1_) followed by the integration circuit and the comparator. The squaring circuits usually take advantage of the squaring feature of MOSFETs [48]. The latter needs the *upward event-driven architecture* where the event-triggered communication is implemented on the top of the background time-triggered signal acquisition [49].

### 2.3. Motivation

The motivation to introduce the energy criterion for event-based sampling is similar to the argumentation for using the integral criteria [8,23]. As stated in [8,23], in the linear send-on-delta algorithm, the signal oscillations or the steady-state error are not detected by the sampling operations if these oscillations remain within the threshold [8,23]. In the sampling according to the error energy criterion, similarly like in the integral sampling schemes, the signal is “tracked” continuously since signal variations are accumulated during the sampling interval.

As follows from (3), in the error energy sampling criterion similarly as in the integral sampling, the sampling time is a function of the intersampling signal behavior since the signal variations that occur within the intersampling interval are successively accumulated [8]. The difference is that the successive sampling error values are squared before integration in the error energy based sampling criterion. Compared to the integral sampling criterion [8] and to the area-triggered sampling [23]), the error energy criterion gives more weight to extreme sampling error values.

Furthermore, the energy is one of fundamental signal measures with an important physical interpretation. The performance of monitoring or control system is usually defined as integral or energy of the error. The sampling according to energy criterion is useful in case of *indirect measurements*, in particular, when the measurements in the energy domain are performed. For example, it occurs if the electric energy is measured by sampling of current intensity signal or if the mechanical energy is estimated on a basis of tracking velocity.

## 3. Analytical Modeling of Sampling according to Energy Criterion

### 3.1. Mean Sampling Rate Approximation

In the event-based schemes, the current sampling rate depends on the input signal variations and the *sampling resolution* defined as 1/*ζ*. Intuitively, the high sampling resolution (small *ζ*) causes the number of samples in a time unit to increase, and conversely. Now we will derive the relationship between the sampling resolution *ζ* (threshold) and the mean rate of samples taken during the specified time interval [*a*,*b*].

By letting the signal *x*(*t*) be approximated by a truncated Taylor series, if the threshold *ζ* is small enough we have:

(4) $$x(t)-x(t_{i-1})≅x^{\prime }(t_{i-1})(t-t_{i-1})\;\text{for}\;t_{i-1}\leq t\leq t_{i}$$

where *x*′(*t*_*i*−1_) is the signal time-derivative at the instant *t*_*i*−1_.

Hence:

(5) $$\xi =\int_{t_{i-1}}^{t_{i-1}+\Delta t_{i}}{\left[x(t)-x(t_{i-1})\right]}^{2}\;\mathrm{dt}≅\int_{0}^{\Delta t_{i}}{\left[x^{\prime }(t_{i-1})\right]}^{2}\;t^{2}\;\mathrm{dt}=\frac{{\left[x^{\prime }(t_{i-1})\right]}^{2}}{3}\;{\left(\Delta t_{i}\right)}^{3}$$

where Δ*t_i_* = *t_i_* − *t*_*i*−1_ is the length of the *i*th sampling interval.

The Δ*t_i_* can be derived from (5) as a function of the signal derivative *x*′(*t*_*i*−1_) at the point *t*_*i*−1_ and the sampling resolution *ζ*:

(6) $$\Delta t_{i}=\sqrt[{3}]{\frac{3\zeta }{{\left[x^{\prime }(t_{i-1})\right]}^{2}}}$$

By the definition, the mean sampling rate *s_L_*, defined as the average number of samples in a time unit, in the uniform sampling in the energy domain is given by:

(7) $$s_{L}\overset{\mathrm{def}}{=}\frac{n}{\sum_{i=1}^{n}\;\Delta t_{i}}$$

For the sake of simplicity but without loss of generality, assume that the analyzed time interval is a sum of the sampling intervals, *i.e.*, *t*_0_ = *a*, *t_n_* = *b*, and:

(8) $$\sum_{i=1}^{n}\Delta t_{i}=b-a$$

Furthermore, let us assume that the signal derivative *x*′(*t*) is constant at any time instant *t* within the *i*th sampling interval (*t*_*i*−1_,*t_i_*) and equal to the derivative at the point *t*_*i*−1_ in the beginning of this interval:

(9) $$\forall t\in \;(t_{i-1},\;t_{i}):x^{\prime }(t)≅x^{\prime }(t_{i-1})$$

Hence:

(10) $$\sum_{i=1}^{n}\;\int_{t_{i-1}}^{t_{i}}\;\sqrt[{3}]{{\left[x^{\prime }(t_{i-1})\right]}^{2}}\;\mathrm{dt}≅\sum_{i=1}^{n}\;\sqrt[{3}]{{\left[x^{\prime }(t_{i-1})\right]}^{2}}\;\Delta t_{i}$$

This approximation (10) is more accurate if the threshold *ζ* is small.

On the basis of the equation (6), we obtain:

(11) $$\sum_{i=1}^{n}\;\int_{t_{i-1}}^{t_{i}}\;\sqrt[{3}]{{\left[x^{\prime }(t_{i-1})\right]}^{2}}\;\mathrm{dt}=\sqrt[{3}]{3\zeta }n$$

On the basis of (8), (10), and (11), we obtain the expression for the mean sampling rate:

(12) $$s_{L}=\frac{\sum_{i=1}^{n}\int_{t_{i-1}}^{t_{i}}\sqrt[{3}]{{\left[x^{\prime }(t_{i-1})\right]}^{2}}\;\mathrm{dt}}{\sqrt[{3}]{3\zeta }(b-a)}$$

If the lengths of the sampling intervals Δ*t_i_* = *t_i_* − *t*_*i*−1_ are small enough, the expression $\sqrt[{3}]{{\left[x^{\prime }(t_{i-1})\right]}^{2}}$ is constant in time so the sum of integrals on the right side of the formula (12) might be approximated by the single integral of the function $\sqrt[{3}]{{\left[x^{\prime }(t)\right]}^{2}}$ taken over the whole time interval [*a*,*b*] ≡ [*t*_0_,*t_n_*]:

(13) $$s_{L}≅\frac{1}{\sqrt[{3}]{3\zeta }}\bar{\sqrt[{3}]{{\left[x^{\prime }(t)\right]}^{2}}}$$

where $\bar{\sqrt[{3}]{{\left[x^{\prime }(t)\right]}^{2}}}$ denotes the mean of the cubic root of the signal derivative square in the time interval [*a*,*b*] as follows:

(14) $$\frac{\int_{a}^{b}\sqrt[{3}]{{\left[x^{\prime }(t)\right]}^{2}}\mathrm{dt}}{b-a}=\bar{\sqrt[{3}]{{\left[x^{\prime }(t)\right]}^{2}}}$$

Summing up, the mean sampling rate according to the uniform event-driven sampling in the energy domain is expressed by two factors:

- the mean of the cubic root of the signal derivative square $\bar{\sqrt[{3}]{{\left[x^{\prime }(t)\right]}^{2}}}$, which is a measure of the sampled signal *x*(*t*),
- the resolution *ζ* of the energy sampling (threshold).

### 3.2. Selection of Sampling Period in Uniform Scheme

To estimate how effective the sampling according to energy criterion is in terms of the sampling rate, we compare it with the conventional periodic scheme. As a criterion of comparison we choose the same *maximum energy of sampling error* in both sampling schemes.

As a *sampling period T* we select such value that the energy of a difference between the current signal value *x*(*t*) and the value included in the most recent sample *x*(*t*_*i*−1_) = *x*((*i*−1)*T*) accumulated during each sampling period cannot exceed a given threshold *ζ* > 0 (compare with (3)):

(15) $$\int_{(i-1)T}^{\mathrm{iT}}{\left[x(t)-x((i-1T))\right]}^{2}\;\mathrm{dt}\leq \zeta$$

The sampling period *T* in the uniform scheme is adjusted to the fastest change of a signal during a time interval [*a*,*b*] so the following relationship is valid:

(16) $$\xi =\int_{0}^{T}{\left[x^{\prime }{\left(t\right)}_{\text{max}}\right]}^{2}\;t^{2}\;\mathrm{dt}=\frac{{\left[x^{\prime }{\left(t\right)}_{\text{max}}\right]}^{2}}{3}\;T^{3}$$

where |*x*′(*t*)|_max_ = max{|*x*′(*t*)|; t ∈ [*a*,*b*]} is the maximum of the first signal derivative in relation to the time during an interval [*a*,*b*].

Taking into account (16), the sampling rate *s_R_* in the periodic scheme is selected according to the following formula:

(17) $$s_{R}\overset{\mathrm{def}}{=}\frac{1}{T}=\sqrt[{3}]{\frac{{\left[x^{\prime }{\left(t\right)}_{\text{max}}\right]}^{2}}{3\zeta }}$$

The energy of the sampling error in the sampling period *T* reaches its maximum *ζ* only if the signal *x*(*t*) changes its value with the maximum rate defined by |*x*′(*t*)|_max_. During slow signal variations, the energy of the sampling error accumulated in the sampling period *T* is lower. Instead, the energy of the sampling error in the sampling scheme according to the energy criterion equals *ζ* in each sampling interval.

### 3.3. Effectiveness of Event-Based Sampling according to Energy Criterion

The effectiveness of event-based sampling according to energy criterion is defined similarly to the classical send-on-delta [1] and the integral sampling schemes [8] as the ratio of the periodic *s_R_* and the mean rate *s_L_* of the event-based sampling according to energy criterion measured during a certain time interval [*a*,*b*]:

(18) $$\chi =\frac{s_{R}}{s_{L}}$$

where the maximum energy of the sampling error *ζ* is the same in both schemes.

By setting (12) and (17) to (18), we have:

(19) $$\chi =\sqrt[{3}]{{\left[x^{\prime }{\left(t\right)}_{\text{max}}\right]}^{2}}\;\frac{\sum_{i=1}^{n}\;\int_{t_{i-1}}^{t_{i}}\sqrt[{3}]{{\left[x^{\prime }(t)\right]}^{2}}\;\mathrm{dt}}{b-a}$$

Since the sampled signal *x*(*t*) is approximated by the first two terms of the Taylor series (see (4)), the formula (19) gives only an approximation of the real sampling effectiveness. This approximation is more accurate if the number of samples, *n*, taken during a specified time interval [*a*,*b*] is high.

In particular, if the sampling resolution is infinite (*ζ*→0), then n→∞, and the sum in the numerator of the formula (20) approaches the integral as follows:

(20) $$\underset{n\to \infty }{\text{lim}}\sum_{i=1}^{n}\;\int_{t_{i-1}}^{t_{i}}\;\sqrt[{3}]{{\left[x^{\prime }(t)\right]}^{2}}\;\mathrm{dt}=\int_{a}^{b}\;\sqrt[{3}]{{\left[x^{\prime }(t)\right]}^{2}}\;\mathrm{dt}$$

Let us define the *asymptotic effectiveness χ*_∞_ of the sampling in energy domain in relation to the periodic sampling as:

(21) $$\chi_{\infty }\overset{\mathrm{def}}{=}\underset{1/\zeta \to \infty }{\text{lim}}\;\chi$$

By setting (19) and (20) to (21), we have:

(22) $$\chi_{\infty }=\underset{1/\zeta \to \infty }{\text{lim}}\chi =\underset{n\to \infty }{\text{lim}}\frac{s_{R}}{s_{L}}=(b-a)\frac{\sqrt[{3}]{{\left[x^{\prime }{\left(t\right)}_{\text{max}}\right]}^{2}}}{\int_{a}^{b}\sqrt[{3}]{{\left[x^{\prime }(t)\right]}^{2}}\;\mathrm{dt}}$$

Finally, taking into (14) in (22), the final formula for the asymptotic effectiveness is obtained:

(23) $$\chi_{\infty }=\frac{\sqrt[{3}]{{\left[x^{\prime }{\left(t\right)}_{\text{max}}\right]}^{2}}}{\sqrt[{3}]{{\left[x^{\prime }(t)\right]}^{2}}}$$

As follows from (23), the asymptotic effectiveness *χ*_∞_ is independent of the sampling resolution defined by *ζ*, and constitutes the embedded feature of the sampled signal similarly as for the send-on-delta scheme [1], and the integral sampling [8].

### 3.5. Comparing Sampling in Energy Domain to Integral Sampling and to Send-on-Delta Scheme

Comparison of the asymptotic effectiveness *χ*_∞_ of sampling according to energy criterion defined by (23) to the asymptotic effectiveness *q*_∞_ of integral sampling [8], and the minimum effectiveness *p*_min_ of the send-on-delta scheme [1] shows that the sampling in energy domain is less effective than the send-on-delta scheme but more effective than integral sampling (for the send-on-delta scheme, the asymptotic effectiveness is the minimum effectiveness at the same time [1]):

(24) $$q_{\infty }\leq \chi_{\infty }\leq p_{\text{min}}$$

where $p_{\text{min}}=\frac{{\left|x^{\prime }(t)\right|}_{\text{max}}}{\left|x^{\prime }(t)\right|}$ and $q_{\infty }=\frac{\sqrt{{\left|x^{\prime }(t)\right|}_{\text{max}}}}{\sqrt{\left|x^{\prime }(t)\right|}}$.

Moreover, the inequality (24) is reduced to the equality (*q*_∞_ = *χ*_∞_ = *p*_min_) only for a pure linear signal. For the other continuous-time signals, the inequality (24) is strong, *i.e.*, *q*_∞_ < *χ*_∞_ < *p*_min_.

On the other hand, it is straightforward to show that higher effectiveness in sampling according to the selected event-based criterion is obtained at the cost of increasing the total sampling error defined as the sum of errors for all the samples taken during a specified time interval.

Thus, the ratio of the sum of energy of all the sampling errors in sampling according to energy criterion to the energy of all the sampling errors in periodic sampling is higher than the ratio of the total integral error in the integral sampling to the total integral error in the periodic sampling. On the other hand, the opposite relationship exists for the sampling according to energy criterion compared to the linear send-on-delta scheme (the ratio of the sum of error energy in sampling in energy domain and periodic sampling is lower than the ratio of the total linear error in send-on-delta scheme and in periodic sampling).

### 3.6. Generalized Event-Based Sampling Criteria

The signal-dependent event-based sampling criteria might be approximated by a unified integral criterion as follows. The continuous-time signal *x*(*t*) is sampled at the instant *t_i_* according to the event-based sampling criterion if the characteristic function *z*[*x*(*t*) − *x*(*t*_*i*−1_)] of a difference between the signal value *x*(*t_i_*) and the value included in the most recent sample *x*(*t*_*i*−1_) accumulated during the *i*th sampling interval (*t*_*i*−1_,*t_i_*) reaches a certain constant threshold *θ* > 0:

(25) $$z[x(t)-x(t_{i-1})]=\frac{1}{{\left(t_{i}-t_{i-1}\right)}^{k}}\int_{t_{i-1}}^{t_{i}}{\left[x(t)-x(t_{i-1})\right]}^{l}\;\mathrm{dt}=\theta$$

where *k*, *l* > 0 are integers.

In particular, the integral sampling scheme is modeled for *k* = 0, *l* = 1, and the sampling according to energy criterion for *k* = 1, *l* = 2, respectively. For *k* = 1, *l* = 1, the generalized sampling criterion approximates the model of the send-on-delta principle for high sampling resolution (*i.e.*, if *θ* is small). More precisely, modeling the send-on-delta scheme by the generalized event-based sampling criterion (25) is accurate provided that the approximation stated by (4) is satisfactory.

The generalized criterion defines also a set of the other event-based sampling criteria, for example, the *time-weighted integral criterion* for *k* = −1, *l* = 1. However, some event-based sampling schemes can be unstable, especially for slowly varying signals and high *θ*, meaning that the events triggering sampling operations might not occur for long time.

The generalized criterion (25) for event-based sampling is similar to the generalized criteria for adaptive sampling formulated in [15,18].

## 4. Event-Based Spatial Sampling

Since most physical phenomena are widespread in time and space, one of the primary objectives of sensor networks is to report the spatial distribution of a particular parameter (e.g., a temperature).

The traditional wired and wireless data and sensor networks have been designed as node-centric since data have been requested from a specific node. The *node-centric* networks are able to gather data from a selected *points of interest* (e.g., a room) where the knowledge of the spatial distribution of a physical magnitude being measured is not important.

Modern sensor networking architecture are *data-centric* rather than node-centric [50], meaning that data is requested based on certain attributes such as those which area has temperature greater than 20 °C [51]. The data-centric networks enable a systematic spatial monitoring of the sensor field. The representative application of spatial sampling in sensor networking is environment monitoring (e.g., fundamental processes of meteorology). Sampling and estimation of geographical attributes that vary across space (e.g., area temperature, urban pollution level) are common in many real-world applications [52,53].

### 4.1. Event-Based Spatial Reporting Strategy

The concept of event-based temporal sampling can be easily extended to sampling signals in the space domain. The difference is that continuous sensing of signals in the space, similarly as may be carried out in the time domain by the use of an analog sampling circuit, is impracticable in general. Instead, the continuous space signals are sampled by sensors deployed at discrete locations. Thus, the *upward event-driven architecture* is recommended to implement the distributed event-based system to sample continuous-space signals (see Section 2.2) [49].

Event-based reporting in the sensor networked system with the upward event-driven architecture is a two-step process corresponding to fine and coarse quantization of the signal value. First, the continuous-space signal is sampled by sensors deployed along the space being monitored (e.g., uniformly every unit distance) which results in conversion of the continuous-space to the discrete-space signal. Next, the sensors process the samples and communicate together in order to detect a significant change of a characteristic parameter defined by the event-based reporting criterion. The specified change of the selected parameter is searched along the one-dimensional space in relation to the selected reference position. The locations of the sensing devices corresponding to the positions where the specified changes have been detected create the (one-dimensional) discrete-space picture of the characteristic parameter distribution. The density of sensors deployment should be high enough referred to the intended resolution of the event-based reporting. The concept of sampling continuously-spaced signals may be extended to two-dimensional and three-dimensional space domains.

### 4.2. Modelling Event-Based Spatial Sampling Density

Let as assume that the signal *x*(*s*) is a function of a one-dimensional space *s*. In order to keep the analysis as general as possible, we assume that continuous sensing of signals in the space domain is practicable. Furthermore, let us assume that the continuous-space signal *x*(*s*) is sampled at the position *s* > *s*_i−1_ if a characteristic parameter *y*[*x*(*s*) − *x*(*s*_*i*−1_)] accumulated along the distance (*s*_*i*−1_,*s*), where *s*_*i*−1_ is a position where the previous sample has been taken, reaches a certain constant threshold *ζ* > 0.

The definition of the characteristic parameter *y*[*x*(*s*) − *x*(*s*_*i*−1_)] depends on the sampling criterion as follows:

- spatial (linear) sampling error [*x*(*s*) − *x*(*s*_*i*−1_)] in the spatial send-on-delta sampling scheme,
- integral of the spatial sampling error $\int_{s_{i-1}}^{s}\;[x(s)-x(s_{i-1})]\mathrm{ds}$ in the spatial integral sampling,
- energy of the spatial sampling error $\int_{s_{i-1}}^{s}\;{\left[x(s)-x(s_{i-1})\right]}^{2}\;\mathrm{ds}$ in the spatial sampling according to the energy criterion.

The mean spatial sampling densities are expressed by the formulae equivalent to that derived for the corresponding temporal sampling criteria. In particular, the mean spatial sampling density *s_L_* according to the error energy criterion depends on the signal space-derivative $\bar{\sqrt[{3}]{{\left[x^{\prime }(s)\right]}^{2}}}$ along the sampling distance on the one hand, and the sampling resolution *ζ* on the other :

(26) $$s_{L}≅\frac{1}{\sqrt[{3}]{3\zeta }}\;\bar{\sqrt[{3}]{{\left[x^{\prime }(s)\right]}^{2}}}$$

where $\bar{\sqrt[{3}]{{\left[x^{\prime }(s)\right]}^{2}}}$ denotes the mean of the cubic root of the signal space-derivative square along the sampling distance [*a*,*b*] as follows:

(27) $$\frac{\int_{a}^{b}\sqrt[{3}]{{\left[x^{\prime }(s)\right]}^{2}}\;\mathrm{dt}}{b-a}=\bar{\sqrt[{3}]{{\left[x^{\prime }(s)\right]}^{2}}}$$

The mean spatial sampling densities according to the send-on-delta and to integral criterion may be defined similarly as for the corresponding event-based temporal schemes, see [1,8].

## 5. Simulation Results

To verify the derived analytical formula (23) for the asymptotic effectiveness of sampling in energy domain, we have run the simulations in Matlab/Simulink environment.

**Figure 2.**
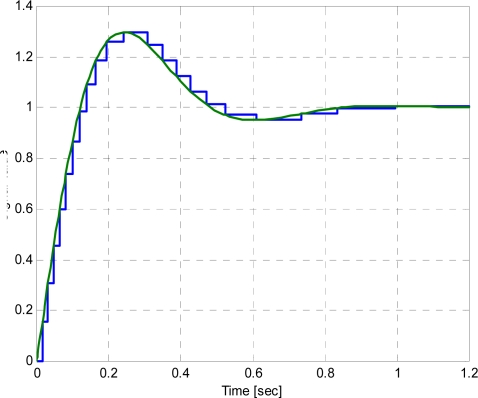
Sampling according to the energy criterion of the test signal.

The test signal *x*(*t*) is the step-response of the second-order underdamped closed-loop system, given by the open-loop transfer function *F*(*s*) = (10*s* + 100)/*s*^2^. The signal *x*(*t*) has the following form in the time domain:

(28) $$x(t)=1+\frac{\sqrt{3}}{3}\;e^{-5t}\;\text{sin}\;5\sqrt{3}t-e^{-5t}\;\text{cos}\;5\sqrt{3}t$$

and is presented in the Figure 2.

**Figure 3.**
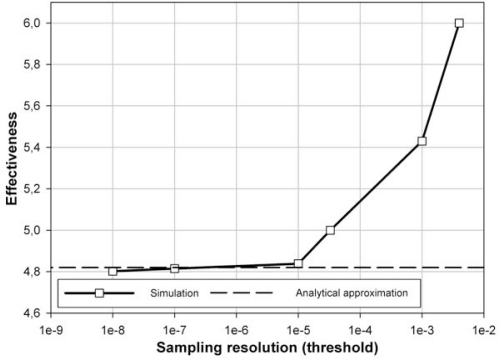
Simulation results and the analytical approximation of the effectiveness of the sampling according to energy criterion for the test signal.

The signal given by (28) has been used by several authors for investigating both the adaptive sampling algorithms and the event-based schemes (*i.e.*, send-on-delta [1] and integral sampling [8]). The simulation has been run for the time interval (0;1.2[sec]) when the transient component of the response dies out and the signal becomes nearly constant at the end of the selected time interval. The zero-order hold is used. The simulation results are presented in Table 1 and in Figure 3.

Let us compare the simulation results of sampling effectiveness with the analytical approximation given by the formula (23). The mean of the cubic root of the signal derivative square $\bar{\sqrt[{3}]{{\left[x^{\prime }(t)\right]}^{2}}}$, computed numerically for the time interval (0;1.2[sec]) equals:

$$\bar{\sqrt[{3}]{{\left[x^{\prime }(t)\right]}^{2}}}=\frac{\int_{0}^{1.2}\;\sqrt[{3}]{{\left(\left|\frac{10}{3}\;e^{-5t}\left(3\;\text{cos}\left[5\sqrt{3}t\right]+\sqrt{3}\;\text{sin}\left[5\sqrt{3}t\right]\right)\right|\right)}^{2}}\;\;\mathrm{dt}}{1.2\sqrt[{3}]{10^{2}}}≅0.948$$

Furthermore:

$$\sqrt[{3}]{{\left[x^{\prime }(t)\right]}_{\text{max}}^{2}}≅\sqrt[{3}]{10^{2}}$$

and

$$\chi_{\infty }≅4.82$$

The asymptotic effectiveness of sampling the test signal according to energy criterion equal to 4.82 is presented in Figure 3 as a horizontal asymptote together with simulation results. The sampling effectiveness for the highest but finite resolution 1/*ζ* = 10^8^ obtained by simulation, equal to 4.802, is very close to the asymptotic value derived analytically.

For comparison, the asymptotic (and minimum) effectiveness of the linear send-on-delta for the test signal equals 7.02 [1], and the asymptotic effectiveness of the sampling according to integral criterion is 3.72 [8].

## 6. Asymptotic Effectiveness of Event-Based Sampling in Dynamic Systems

On the basis of the formula (23), the analytic expressions for the asymptotic effectiveness of the sampling in the energy domain for particular step responses of the first and second-order dynamic systems are listed. The signals defined by these step responses model a temporal evolution of the controlled object in many sensory applications.

The asymptotic effectiveness of the following signals are estimated:

- step responses of the first-order system, of the differentiation circuit, and of the integration circuit,
- critically damped step responses of the second-order and the *n*th-order systems,
- second-order overdamped step response,
- undamped step response (harmonic signal).

The results are shown in Table 2. See for comparison corresponding results of the send-on-delta and integral sampling effectiveness listed in [1] and in [8].

As follows from Table 2, the asymptotic effectiveness of the sampling according to energy criterion for the time interval [0,*b*] is a function of *η* (or *η*_1_ and *η*_2_ for the system with different time constants), which is the length of the considered time interval (*b*) normalized to the appropriate time constants (*η* = *b*/*T*, *η*_1_ = *b*/*T*_1_, *η*_2_ = *b*/*T*_2_). Note that the effectiveness for the pure harmonic signal equals 1.4 (for a comparison, the effectiveness of the linear send-on-delta equal 1.57, and of the integral criterion 1.31, respectively).

In Table 3, the numerical values of the asymptotic effectiveness of the sampling according to energy criterion are listed. The time interval [0,*b*] selection (except for a step response of the undamped system) is based on the assumption that *b* represents the *settling time* of a system, *i.e.*, the time required for the step response to stay within a specified percentage of its steady-state value. The percentage is shown in the first column, e.g., for the second-order critically damped system, *b* = 5*T* is set, because *x*(5*T*) = 0.959*x*_0_ where *x*_0_ is the steady-state value. For the second-order overdamped system, *T*_1_/*T*_2_ = 7/5 is assumed since *x*(5*T*_1_ = 7*T*_2_) = 0.97*x*_0_.

The presented results show that the asymptotic effectiveness of sampling in energy domain ranges between 1.039 and 2.31 for signals selected in Table 3. Only the effectiveness for the pure linear signal equals 1.

For the send-on-delta scheme, the asymptotic effectiveness is the minimum effectiveness at the same time, and in particular, the effectiveness of the send-on-delta scheme for the non-monotonic signals is independent of the sampling resolution [1]. Thus, for non-monotonic signals, the send-on-delta effectiveness is denoted by *p* instead of *p*_min_ in Table 3.

## 7. Conclusions

The present paper is a next step in the series of the studies that deal with the effectiveness of the particular event-based sampling scheme compared to the conventional periodic sampling as a reference. The comparison of both schemes is carried out for equality of the maximum sampling error in the domain that corresponds to the particular event-based sampling criterion. In the present study, the event-based sampling scheme according to a constant energy of sampling error is analyzed. As expected, this criterion is suitable for applications where the energy of sampling error should be bounded. Thus, the proposed sampling scheme extends a range of event-based sampling schemes and makes the choice of particular sampling criterion more flexible to application requirements.

In the paper, it is proved analytically that in order to keep the energy of sampling error bounded, the proposed event-based sampling criterion is more effective than the periodic sampling by a factor defined by the signal derivative. Furthermore, it is shown that the sampling according to energy criterion is less effective than the send-o-delta scheme but more effective than the sampling according to integral criterion. On the other hand, it is indicated that higher effectiveness in sampling according to the selected event-based criterion is obtained at the cost of increasing the total sampling error defined as the sum of errors for all the samples taken during a specified time interval.

## Figures and Tables

**Table 1.** Simulation results for the energy sampling effectiveness for the test signal.

Sampling resolution (threshold *ζ*)	Number of samples in energy sampling	Number of samples in periodic sampling	Energy sampling effectiveness
4E-3	4	24	**6**
1E-3	7	38	**5.430**
3.3E-5	24	120	**5**
1E-5	37	179	**4.838**
1E-7	173	833	**4.815**
1E-8	373	1,791	**4.802**

**Table 2.** The asymptotic effectiveness of event-based sampling in energy domain for step responses of selected dynamic systems for the time interval [0,*b*] where *η* = *b*/*T*, *η*_1_ = *b*/*T*_1_, *η*_2_ = *b*/*T*_2_.

**Signal**	**Step response**	**Asymptotic effectiveness**
$x(t)=k(1-e^{-\frac{t}{T}})$	First-order step response	$\chi_{\infty }=\frac{2}{3}\;\frac{\lambda }{1-e^{-\frac{2}{3}\lambda }}$
$x(t)=\frac{k}{T}\;e^{-\frac{t}{T}}$	Differentiation circuit	$\chi_{\infty }=\frac{2}{3}\;\frac{\lambda }{1-e^{-\frac{2}{3}\lambda }}$
$x(t)=\mathrm{kt}-\mathrm{kT}(1-e^{-\frac{t}{T}})$	Integration circuit	χ∞=2⋅(1−e−λ)23⋅λ3⋅(1−e−λ)23+2⋅(−1)13⋅(2π3−Beta[eλ,13, 23])
$x(t)=k[1-(1+\frac{t}{T})e^{-\frac{t}{T}}]$	Second-order critically damped step response	χ∞=2e23(−1+λ) λλ23+(32eλ)23(−Gamma[53]+23Gamma[23, 2λ3])
$x(t)=k(1+\frac{T_{1}}{T_{2}-T_{1}}e^{-\frac{t}{T_{1}}}-\frac{T_{2}}{T_{2}-T_{1}}e^{-\frac{t}{T_{2}}})$	Second-order overdamped step response	χ∞=23(2)23(1λ1(1−e−2λ13)+1λ2(1−e−2λ23))
$x(t)=1-e^{-\frac{t}{T}}\sum_{k=0}^{n-1}\;\frac{{\left(\frac{t}{T}\right)}^{k}}{k!}$	*n*th-order critically damped step response	$\chi_{\infty }=\frac{{\left(\frac{3}{2}\right)}^{-\frac{2n+1}{3}}\;\lambda {\left(\left(e^{1-n}\right){\left(n-1\right)}^{n-1}\right)}^{\frac{2}{3}}}{\mathrm{Gamma}\left[\frac{2n+1}{3}\right]-\mathrm{Gamma}\left[\frac{2n+1}{3},\;\frac{2\lambda }{3}\right]}$
$\begin{array}{c} x(t)=k[1-\frac{e^{-\omega_{n}\xi t}}{\sqrt{1-\xi^{2}}}\;\text{sin}(\omega_{0}t+\gamma )]\\ \text{where}:\\ \omega_{0}=\omega_{n}\sqrt{1-\xi^{2}};\;\gamma =\text{arc tg}\sqrt{1-\xi^{2}}/\xi \end{array}$	Second-order underdamped step response (0 < *ξ* < 1)	Symbolic solution is not available, the numeric solutions for a particular set of parameters can be calculated
*x*(*t*) = *k*(1 − cos *ω_n_ t*)	Harmonic signal—second-order undamped step response (*ξ* = 0)	$\begin{array}{c} \chi_{\infty }=\frac{2\cdot \pi \cdot \mathrm{Gamma}\left[\frac{4}{3}\right]}{\sqrt{\pi }\cdot \mathrm{Gamma}\left[\frac{5}{6}\right]-\mathrm{Beta}\left[1,\;\frac{1}{2},\;\frac{5}{6}\right]\cdot \sqrt{{\text{cos}}^{2}\pi }\cdot \mathrm{Gamma}\left[\frac{4}{3}\right]\cdot \text{sec}\;\pi }=1,4\;;\\ b=m\pi /2\omega_{n}\;\;;\;\;m=\ldots -1,0,1,2,\ldots \end{array}$

where: $\mathrm{Gamma}(n)=\int_{0}^{\infty }t^{n-1}\;e^{-t}\;\mathrm{dt}$ - the *Gamma function*, $\mathrm{Gamma}(n,\;\lambda )=\int_{\lambda }^{\infty }t^{n-1}\;e^{-t}\;\mathrm{dt}$- the *incomplete Gamma function*, $\mathrm{Beta}(z,\;a,\;b)=\int_{0}^{z}t^{a-1}\;{\left(1-t\right)}^{b-1}\;\mathrm{dt}$ - the *incomplete Beta function*.

**Table 3.** Numerical values of asymptotic effectiveness of event-based sampling according to energy criterion for selected time intervals [0,*b*].

**Signal**	**Energy sampling effectiveness**	**Integral sampling effectiveness**	**Send-on-delta effectiveness**
$\begin{array}{c} x(t)=x_{0}(1-e^{-\frac{t}{T}})\\ x(3T)=0.95x_{0} \end{array}$	*χ*_∞_(*η* = 3) = **2,31** where: *η* = *b*/*T*	*q_∞_*(*η* = 3) = **1.93**	*p*(*η* = 3) = **3.16**
$\begin{array}{c} x(t)=\frac{x_{0}}{T}\;e^{-\frac{t}{T}}\\ x(3T)=0,05x_{0}/T \end{array}$	*χ*_∞_(*η* = 3) = **2,31** where: *η* = *b*/*T*	*q*_∞_(*η* = 3) = **1.93**	*p*(*η* = 3) = **3.16**
$\begin{array}{c} x(t)=x_{0}t-x_{0}T(1-e^{-\frac{t}{T}})\\ x(20T)=0,95\cdot x_{0}t \end{array}$	*χ*_∞_(*η* = 20) = **1.039** where: *η* = *b*/*T*	*q*_∞_(*η* = 20) = **1.032**	*p*(*η* = 20) = **1.053**
$\begin{array}{c} x(t)=x_{0}[1-(1+\frac{t}{T})e^{-\frac{t}{T}}]\\ x(5T)=0.959x_{0} \end{array}$	*χ*_∞_(*η* = 20) = **1.85** where: *η* = *b*/*T*	*q*_∞_(*η* = 20) = **1.46**	*p*(*η* = 20) = **1.92**
$\begin{array}{c} x(t)=1-e^{-\frac{t}{T}}\sum_{k=0}^{n-1}\;\frac{{\left(\frac{t}{T}\right)}^{k}}{k!}\\ x(n=2,\;\eta =5)=0.96x_{0} \end{array}$	*χ*_∞_(*n* = 2, *λ* = 5) = **1,62** where: *η* = *b*/*T*	*q*_∞_(*n* = 2,*η* = 5) = **1.46**	*p*(*n* = 2, *λ* = 5) = **1,92**
*x* (*t*) = *x*_0_ (1 − cos *ω_n_ t*)	*χ*_∞_ = **1,402** *bω_n_* = *πm*/2, *m* = 1,2,…	*q*_∞_ = **1.31**	*p*_min_ = **1.57**
*x*(*t*) = *kt*	*χ*_∞_ = **1**	*q*_∞_ = **1**	*p* = **1**

## References

[1] Miśkowicz M. (2006). Send-on-Delta Concept: an Event-Based Data Reporting Strategy. Sensors.

[2] Pawlowski A., Guzman J.L., Rodríguez F., Berenguel M., Sánchez J., Dormido S. (2009). Simulation of Greenhouse Climate Monitoring and Control with Wireless Sensor Network and Event-Based Control. Sensors.

[3] Allier E., Sicard G., Fesquet L., Renaudin M. A New Class of Asynchronous A/D Converters Based on Time Quantization.

[4] Manohar R., Apsel A.B., Akopyan F. A Level-Crosing Flash Asynchronous Analog-to-Digital Converter.

[5] Kościelnik D., Miśkowicz M. (2008). Asynchronous Sigma-Delta Analog-to-Digital Converter Based on the Charge Pump Integrator. Analog Integr. Circuit. Sig.

[6] MalmirChegini M., Marvasti F. Performance Improvement of Level-Crossing A/D Converters.

[7] Guan K.M., Kozat S.S., Singer A.C. (2008). Adaptive Reference Levels in a Level-Crossing Analog-to-Digital Converter. EURASIP J. Adv. Sign. Proc.

[8] Miśkowicz M. (2007). Asymptotic Effectiveness of the Event-Based Sampling according to the Integral Criterion. Sensors.

[9] Suh Y.S. (2007). Send-on-Delta Sensor Data Transmission with a Linear Predictor. Sensors.

[10] Ellis P.H. (1959). Extension of Phase Plane Analysis to Quantized Systems. IRE Trans. Automat. Contr.

[11] Dorf R.C., Farren M.C., Phillips C.A. (1962). Adaptive Sampling for Sampled-Data Control Systems. IEEE Trans. Automat. Contr.

[12] Mitchell J.R., McDaniel W.L. (1969). Adaptive Sampling Technique. IEEE Trans. Automat. Contr.

[13] Gupta S.C. (1963). Increasing the Sampling Efficiency for a Control System. IEEE Trans. Automat. Contr.

[14] Hsia T.C. (1972). Comparisons of Adaptive Sampling Control Laws. IEEE Trans. Automat. Contr.

[15] Hsia T.C. (1974). Analytic Design of Adaptive Sampling Control Laws. IEEE Trans. Automat. Contr.

[16] De la Sen M., Almansa A. (2002). Adaptive Stable Control of Manipulators with Improved Adaptation Transients by Using On-Line Supervision of the Free-Parameters of the Adaptation Algorithm and Sampling Rate. Inform. Lith. Acad. Sci.

[17] De la Sen M. (1996). Non-Periodic and Adaptive Sampling. A Tutorial Review. Inform. Lith. Acad. Sci.

[18] Dormido S., de la Sen M., Mellado M. (1978). Criterios Generales de Determinación de Leyes de Maestro Adaptivo (in Spanish). Revista de Informática y Automática.

[19] Xia F., Zhao W. (2007). Flexible Time-Triggered Sampling in Smart Sensor-Based Wireless Control Systems. Sensors.

[20] Henriksson D., Cervin A. Optimal On-line Sampling Period Assignment for Real-Time Control Tasks Based on Plant State Information.

[21] Manjeshwar A., Agrawal D.P. TEEN: A Routing Protocol for Enhanced Efficiency in Wireless Sensor Networks.

[22] Nguyen V.H., Suh Y.S. (2009). Networked Estimation for Event-Based Sampling Systems with Packet Dropouts. Sensors.

[23] Nguyen V.H., Suh Y.S. (2008). Networked Estimation with an Area-Triggered Transmission Method. Sensors.

[24] Vasyutynskyy V., Kabitzsch K. Towards Comparison of Deadband Sampling Types.

[25] Ploennigs J., Vasyutynskyy V., Kabitzsch K. Comparison of Energy-Efficient Sampling Methods for WSNs in Building Automation Scenarios.

[26] Sánchez J., Guarnes M.Á., Dormido S. (2009). On the Application of Different Event-Based Sampling Strategies to the Control of a Simple Industrial Process. Sensors.

[27] de la Sen M. (2007). About Optimal Fractional Hold Circuits for Inter-Sample Output Reconstruction in Sampled-Data Systems. Sensors.

[28] de la Sen M. (2007). On the Properties Of Reachability, Observability, Controllability, and Constructibility of Discrete-Time Positive Time-Invariant Linear Systems with Aperiodic Choice of the Sampling Instants. Disc. Dynam. Nat. Soc.

[29] (2002). Layer 7 LonMark Interoperability Guidelines.

[30] Miśkowicz M., Golański R. (2006). LON Technology in Wireless Sensor Networking Applications. Sensors.

[31] Neugebauer M., Kabitzsch K. A New Protocol for a Low Power Sensor Network.

[32] Plönnigs J., Neugebauer M., Kabitzsch K. A Traffic Model for Networked Devices in the Building Automation.

[33] Aström K.J., Bernhardsson B., Rantzer A., Byrnes C.I. (2003). Systems with Lebesgue Sampling. Directions in Mathematical Systems Theory and Optimization.

[34] Sayiner N., Sorensen H.V., Viswanathan T.R. (1996). A Level-Crossing Sampling Scheme for A/D Conversion. IEEE Trans. Circuits Syst. II Analog Digit. Sig. Proc.

[35] Mark J., Todd T. (1981). A Nonuniform Sampling Approach to Data Compression. IEEE Trans. Commun.

[36] Otanez P., Moyne J., Tilbury D. Using Deadbands To Reduce Communication in Networked Control Systems.

[37] Cogill R. Event-Based Control Using Quadratic Approximate Value Functions.

[38] Cogill R., Lall S., Hespanha J.P. A Constant Factor Approximation Algorithm for Optimal Estimation Subject to Communication Costs.

[39] Kofman E., Braslavsky J.H. Level Crossing Sampling in Feedback Stabilization under Data-Rate Constraints.

[40] Vasyutynskyy V. (2009). Send-on-Delta-Abtastung in PID-Regelungen.

[41] Vasyutynskyy V., Kabitzsch K. Deadband Sampling in PID Control.

[42] Vasyutynskyy V., Kabitzsch K. Implementation of PID Controller with Send-On-Delta Sampling.

[43] Cervin A., Aström K.J. On Limit Cycles in Event-Based Control Systems.

[44] Zhang G., Zheng W.X. (2009). Stability and Bifurcation Analysis of a Class of Networked Dynamical Systems. IEEE Trans. Circuits Syst–II.

[45] Miśkowicz M. Sampling of Signals in Energy Domain.

[46] Riesz F., Nagy B. (1990). Functional Analysis.

[47] Rudin W. (1987). Real and Complex Analysis.

[48] Sakul C. A New CMOS Squaring Circuit Using Voltage/Current Input.

[49] De Paoli F., Tisato F. (1996). On the Complementary Nature of Event-Driven and Time-Driven Models. Contr. Eng. Pract.

[50] Miśkowicz M. (2006). Comparison of Intensive and Extensive Sensor Networking Technologies. Int. J. Online Eng.

[51] Elson J., Estrin D. (2000). An Address-Free Architecture for Dynamic Sensor Networks.

[52] Wang J.-F., Li L.-F., Christakos G. (2009). Sampling and Kriging Spatial Means: Efficiency and Conditions. Sensors.

[53] Wang J.F., Christakos G., Hu M.G. (2009). Modeling Spatial Means of Surfaces with Stratified Non-Homogeneity. IEEE Trans. Geosc. Rem. Sens.

